# Supplementary material for: Osteocalcin expressing cells from tendon sheaths in mice contribute to tendon repair by activating Hedgehog signaling
Source: eLife. 2017 Dec 15;6:e30474. doi: 10.7554/eLife.30474 (PMC5731821; doi:10.7554/eLife.30474)
Supplement: Figure 1—source data 1. [file elife-30474-fig1-data1.docx]

**Figure 1 – source data 1.** Source data relating to Figure 1B. QRT-PCR analysis of sheath specific markers *Tppp3* and *Bglap* using 2-month-old mouse sheath and tendon tissues with expression normalized to *β-tubulin* and the sheath tissues. n=3 biological replicates per group. Statistical comparisons were performed using a two-tailed Student’s t-test in GraphPad Prism (GraphPad Software, California, USA). s.e.m= standard error of the mean.

| Gene | **Sheath tissue** | s.e.m | **Tendon tissue** | s.e.m | P-value | P-value summary |
| --- | --- | --- | --- | --- | --- | --- |
| *Tppp3* | 1.04 | 0.20 | 0.16 | 0.03 | 0.013 | * |
| *Bglap* | 1.01 | 0.10 | 0.17 | 0.04 | 0.0014 | ** |
