# Supplementary material for: Osteocalcin expressing cells from tendon sheaths in mice contribute to tendon repair by activating Hedgehog signaling
Source: eLife. 2017 Dec 15;6:e30474. doi: 10.7554/eLife.30474 (PMC5731821; doi:10.7554/eLife.30474)
Supplement: Figure 1—source data 2. [file elife-30474-fig1-data2.docx]

**Figure 1 – source data 2.** Source data relating to Figure 1E. QRT-PCR analysis of sheath specific markers *Tppp3* and *Bglap* using sorted primary sheath and tendon cells isolated from the *BGLAP-Cre;Rosa26^mT/mG^* mice with expression normalized to *Gapdh* and the sheath cells. n=3 biological replicates per group. Statistical comparisons were performed using a two-tailed Student’s t-test in GraphPad Prism (GraphPad Software, California, USA). s.e.m= standard error of the mean.

| **Gene** | **Sheath cells** | s.e.m | **Tendon cells** | s.e.m | P-value | P-value summary |
| --- | --- | --- | --- | --- | --- | --- |
| *Tppp3* | 1.01 | 0.11 | 0.20 | 0.02 | 0.0019 | ** |
| *Bglap* | 1.02 | 0.15 | 0.22 | 0.01 | 0.0062 | ** |
