# Supplementary material for: Osteocalcin expressing cells from tendon sheaths in mice contribute to tendon repair by activating Hedgehog signaling
Source: eLife. 2017 Dec 15;6:e30474. doi: 10.7554/eLife.30474 (PMC5731821; doi:10.7554/eLife.30474)
Supplement: Figure 1—figure supplement 1—source data 1. [file elife-30474-fig1-figsupp1-data1.docx]

**Figure 1 figure supplement 1 – source data 1.** Source data relating to Figure 1 figure supplement 1B. The number of colonies was counted in colony formation assay using sheath-derived cells (GFP^+^) isolated from the *BGLAP-Cre;Rosa26^mT/mG^* mice with two different initial cell seeding densities of 1000 or 2000 respectively. n=5 biological replicates per group. s.e.m= standard error of the mean.

| Initial cell seeding density(/60mm dish) | **The number of colonies** | s.e.m |
| --- | --- | --- |
| 1000 | 14.20 | 1.69 |
| 2000 | 31.00 | 3.44 |
