# Supplementary material for: Osteocalcin expressing cells from tendon sheaths in mice contribute to tendon repair by activating Hedgehog signaling
Source: eLife. 2017 Dec 15;6:e30474. doi: 10.7554/eLife.30474 (PMC5731821; doi:10.7554/eLife.30474)
Supplement: Figure 2—source data 1. [file elife-30474-fig2-data1.docx]

**Figure 2 – source data 1.** Source data relating to Figure 2D. QRT-PCR analysis of osteogenesis markers using sorted primary sheath cells isolated from the *BGLAP-Cre;Rosa26^mT/mG^* mice with expression normalized to *Gapdh* and the undifferentiated condition. Undiff. indicates undifferentiated condition. Diff. indicates differentiated condition. n=3 biological replicates per group. Statistical comparisons were performed using a two-tailed Student’s t-test in GraphPad Prism (GraphPad Software, California, USA). s.e.m= standard error of the mean.

| Gene | **Undiff.** | s.e.m | **Diff.** | s.e.m | P-value | P-value summary |
| --- | --- | --- | --- | --- | --- | --- |
| *Bglap* | 1.03 | 0.17 | 12.43 | 1.33 | 0.0010 | *** |
| *Dmp1* | 1.02 | 0.15 | 1337 | 110.6 | 0.0003 | *** |
| *Ibsp1* | 1.03 | 0.17 | 99.57 | 11.12 | 0.0009 | *** |
| *Mepe* | 1.02 | 0.12 | 20.81 | 2.26 | 0.0009 | *** |
