# Supplementary material for: Osteocalcin expressing cells from tendon sheaths in mice contribute to tendon repair by activating Hedgehog signaling
Source: eLife. 2017 Dec 15;6:e30474. doi: 10.7554/eLife.30474 (PMC5731821; doi:10.7554/eLife.30474)
Supplement: Figure 2—source data 2. [file elife-30474-fig2-data2.docx]

| Gene | **Undiff.** | s.e.m | **Diff.** | s.e.m | P-value | P-value summary |
| --- | --- | --- | --- | --- | --- | --- |
| *Pparγ* | 1.02 | 0.14 | 10.09 | 1.14 | 0.0014 | ** |
| *Cebpa* | 1.01 | 0.12 | 8.40 | 1.12 | 0.0028 | ** |
| *Fabp4* | 1.02 | 0.13 | 132 | 13.60 | 0.0007 | *** |
| *Lpl* | 1.02 | 0.16 | 58.88 | 10.50 | 0.0053 | ** |

**Figure 2 – source data 2.** Source data relating to Figure 2F. QRT-PCR analysis of adipogenesis markers using sorted primary sheath cells isolated from the *BGLAP-Cre;Rosa26^mT/mG^* mice with expression normalized to *Gapdh* and the undifferentiated condition. Undiff. indicates undifferentiated condition. Diff. indicates differentiated condition. n=3 biological replicates per group. Statistical comparisons were performed using a two-tailed Student’s t-test in GraphPad Prism (GraphPad Software, California, USA). s.e.m= standard error of the mean.
