# Supplementary material for: Osteocalcin expressing cells from tendon sheaths in mice contribute to tendon repair by activating Hedgehog signaling
Source: eLife. 2017 Dec 15;6:e30474. doi: 10.7554/eLife.30474 (PMC5731821; doi:10.7554/eLife.30474)
Supplement: Figure 2—source data 3. [file elife-30474-fig2-data3.docx]

| Gene | **Undiff.** | s.e.m | **Diff.** | s.e.m | P-value | P-value summary |
| --- | --- | --- | --- | --- | --- | --- |
| *Sox9* | 1.02 | 0.13 | 2.51 | 0.27 | 0.0074 | ** |
| *Aggrecan* | 1.02 | 0.15 | 5.17 | 0.69 | 0.0042 | ** |
| *Col2a1* | 1.03 | 0.17 | 63.74 | 6.92 | 0.0008 | *** |

**Figure 2 – source data 3.** Source data relating to Figure 2H. QRT-PCR analysis of chondrogenesis markers using sorted primary sheath cells isolated from the *BGLAP-Cre;Rosa26^mT/mG^* mice with expression normalized to *Gapdh* and the undifferentiated condition. Undiff. indicates undifferentiated condition. Diff. indicates differentiated condition. n=3 biological replicates per group. Statistical comparisons were performed using a two-tailed Student’s t-test in GraphPad Prism (GraphPad Software, California, USA). s.e.m= standard error of the mean.
