# Supplementary material for: Osteocalcin expressing cells from tendon sheaths in mice contribute to tendon repair by activating Hedgehog signaling
Source: eLife. 2017 Dec 15;6:e30474. doi: 10.7554/eLife.30474 (PMC5731821; doi:10.7554/eLife.30474)
Supplement: Figure 2—figure supplement 1—source data 1. [file elife-30474-fig2-figsupp1-data1.docx]

| Gene | **Control** | s.e.m | **Bmp2** | s.e.m | P-value | P-value summary | **TGF-β1** | s.e.m | P-value | P-value summary |
| --- | --- | --- | --- | --- | --- | --- | --- | --- | --- | --- |
| *Mkx* | 1.04 | 0.19 | 3.01 | 0.19 | 0.0018 | ** | 3.50 | 0.41 | 0.0055 | ** |
| *Scx* | 1.09 | 0.28 | 3.03 | 0.20 | 0.0047 | ** | 6.66 | 0.41 | 0.0004 | *** |

**Figure 2 figure supplement 1– source data 1.** Source data relating to Figure 2 figure supplement 1A. QRT-PCR analysis of tendon progenitor markers *Mkx* and *Scx* using sorted sheath cells isolated from the *BGLAP-Cre;Rosa26^mT/mG^* Tibialis anterior tendon sheath tissues treated with 100ng/ml Bmp2 or 2ng/ml TGF-β1 normalized to *β-tubulin* and the control group. n=3 biological replicates per group. Statistical comparisons were performed using a two-tailed Student’s t-test in GraphPad Prism (GraphPad Software, California, USA). s.e.m= standard error of the mean.
