# Supplementary material for: Osteocalcin expressing cells from tendon sheaths in mice contribute to tendon repair by activating Hedgehog signaling
Source: eLife. 2017 Dec 15;6:e30474. doi: 10.7554/eLife.30474 (PMC5731821; doi:10.7554/eLife.30474)
Supplement: Figure 2—figure supplement 1—source data 3. [file elife-30474-fig2-figsupp1-data3.docx]

| Gene | **Control** | s.e.m | **Bmp2** | s.e.m | P-value | P-value summary | **TGF-β1** | s.e.m | P-value | P-value summary |
| --- | --- | --- | --- | --- | --- | --- | --- | --- | --- | --- |
| *Sox9* | 1.00 | 0.05 | 1.76 | 0.09 | 0.0020 | ** | 2.30 | 0.21 | 0.0043 | ** |
| *Col2a1* | 1.02 | 0.15 | 4.97 | 0.38 | 0.0006 | *** | 1.57 | 0.09 | 0.0369 | * |

**Figure 2 figure supplement 1– source data 3.** Source data relating to Figure 2 figure supplement 1C. QRT-PCR analysis of chondrogenic cell lineage markers *Sox9* and *Col2a1* using sorted sheath cells isolated from the *BGLAP-Cre;Rosa26^mT/mG^* Tibialis anterior tendon sheath tissues treated with 100ng/ml Bmp2 or 2ng/ml TGF-β1 normalized to *β-tubulin* and the control group. n=3 biological replicates per group. Statistical comparisons were performed using a two-tailed Student’s t-test in GraphPad Prism (GraphPad Software, California, USA). s.e.m= standard error of the mean.
