# Supplementary material for: Osteocalcin expressing cells from tendon sheaths in mice contribute to tendon repair by activating Hedgehog signaling
Source: eLife. 2017 Dec 15;6:e30474. doi: 10.7554/eLife.30474 (PMC5731821; doi:10.7554/eLife.30474)
Supplement: Figure 3—source data 1. [file elife-30474-fig3-data1.docx]

**Figure 3 – source data 1.** Source data relating to Figure 3E. QRT-PCR analysis of tendon progenitor markers *Mkx* and *Scx* using the Achilles tendon tissues of immunocompromised mice at Day 14 after injury. Sheath transplantation represents transplantation with GFP^+^ sheath-derived cells sorted from the *BGLAP-Cre;Rosa26^mT/mG^* mice with expression normalized to *Gapdh* and the sham group. n=4 biological replicates per group. One-way analysis of variance (ANOVA) followed by Tukey’s tests was used for multiple groups’ comparison in GraphPad Prism (GraphPad Software, California, USA). s.e.m= standard error of the mean.

**Descriptive statistics:**

| Gene | **Sham** | s.e.m | **Injured** | s.e.m | **Injured+ sheath transplantation** | s.e.m |
| --- | --- | --- | --- | --- | --- | --- |
| *Mkx* | 1.02 | 0.13 | 1.33 | 0.04 | 1.77 | 0.12 |
| *Scx* | 1.02 | 0.13 | 0.94 | 0.05 | 1.33 | 0.03 |

**Tukey's multiple comparisons test (Adjusted P Value):**

|  | *Mkx* | Scx |
| --- | --- | --- |
| Sham Vs. Injured | 0.1610 | 0.7768 |
| Sham Vs. Injured+ sheath transplantation | 0.0021 | 0.0621 |
| Injured Vs. Injured+ sheath transplantation | 0.0429 | 0.0213 |
