# Supplementary material for: Osteocalcin expressing cells from tendon sheaths in mice contribute to tendon repair by activating Hedgehog signaling
Source: eLife. 2017 Dec 15;6:e30474. doi: 10.7554/eLife.30474 (PMC5731821; doi:10.7554/eLife.30474)
Supplement: Figure 3—source data 2. [file elife-30474-fig3-data2.docx]

**Figure 3 – source data 2.** Source data relating to Figure 3F. QRT-PCR analysis of tendon ECM component *Col1a1, Col1a2, Tnmd* and *Thbs4* using the Achilles tendon tissues of immunocompromised mice at Day 14 after injury. Sheath transplantation represents transplantation with GFP^+^ sheath-derived cells sorted from the *BGLAP-Cre;Rosa26^mT/mG^* mice with expression normalized to *Gapdh* and the sham group. n=4 biological replicates per group. One-way analysis of variance (ANOVA) followed by Tukey’s tests was used for multiple groups’ comparison in GraphPad Prism (GraphPad Software, California, USA). s.e.m= standard error of the mean.

**Descriptive statistics:**

| Gene | **Sham** | s.e.m | **Injured** | s.e.m | **Injured+ sheath transplantation** | s.e.m |
| --- | --- | --- | --- | --- | --- | --- |
| *Col1a1* | 1.01 | 0.09 | 0.94 | 0.14 | 4.96 | 0.50 |
| *Col1a2* | 1.04 | 0.16 | 0.95 | 0.16 | 2.72 | 0.32 |
| *Tnmd* | 1.01 | 0.09 | 1.77 | 0.18 | 4.45 | 0.67 |
| *Thbs4* | 1.02 | 0.12 | 1.31 | 0.11 | 3.65 | 0.54 |

**Tukey's multiple comparisons test (Adjusted P Value):**

|  | *Col1a1* | *Col1a2* | *Tnmd* | *Thbs4* |
| --- | --- | --- | --- | --- |
| Sham Vs. Injured | 0.9858 | 0.9557 | 0.4128 | 0.8166 |
| Sham Vs. Injured+ sheath transplantation | <0.0001 | 0.0013 | 0.0005 | 0.0008 |
| Injured Vs. Injured+ sheath transplantation | <0.0001 | 0.0009 | 0.0029 | 0.0018 |
