# Supplementary material for: Osteocalcin expressing cells from tendon sheaths in mice contribute to tendon repair by activating Hedgehog signaling
Source: eLife. 2017 Dec 15;6:e30474. doi: 10.7554/eLife.30474 (PMC5731821; doi:10.7554/eLife.30474)
Supplement: Figure 3—source data 3. [file elife-30474-fig3-data3.docx]

**Figure 3 – source data 3.** Source data relating to Figure 3G. Hydroxyproline assay analysis using the Achilles tendon tissues of immunocompromised mice at 4 weeks after injury. Sheath transplantation represents transplantation with GFP^+^ sheath-derived cells sorted from the *BGLAP-Cre;Rosa26^mT/mG^* mice. n=5 biological replicates per group. One-way analysis of variance (ANOVA) followed by Tukey’s tests was used for multiple groups’ comparison in GraphPad Prism (GraphPad Software, California, USA). s.e.m= standard error of the mean.

**Descriptive statistics:**

|  | **Sham (ug/mg tendon)** | s.e.m | **Injured (ug/mg tendon)** | s.e.m | **Injured+ sheath transplantation (ug/mg tendon)** | s.e.m |
| --- | --- | --- | --- | --- | --- | --- |
| Quantity of collagen content | 7.20 | 0.16 | 3.31 | 0.23 | 4.58 | 0.11 |

**Tukey's multiple comparisons test (Adjusted P Value):**

|  | Adjusted P Value | Adjusted P Value summary |
| --- | --- | --- |
| Sham Vs. Injured | <0.0001 | *** |
| Sham Vs. Injured+ sheath transplantation | <0.0001 | *** |
| Injured Vs. Injured+ sheath transplantation | 0.0007 | *** |
