# Supplementary material for: Osteocalcin expressing cells from tendon sheaths in mice contribute to tendon repair by activating Hedgehog signaling
Source: eLife. 2017 Dec 15;6:e30474. doi: 10.7554/eLife.30474 (PMC5731821; doi:10.7554/eLife.30474)
Supplement: Figure 3—source data 4. [file elife-30474-fig3-data4.docx]

**Figure 3 – source data 4.** Source data relating to Figure 3H & 3I. Parameters of mechanical testing of Achilles tendon tissues in the sham, tendon injured and tendon injured with the *BGLAP-Cre;Rosa26^mT/mG^* sheath transplantation groups at 4 weeks after surgery. N≥8 biological replicates per group. One-way analysis of variance (ANOVA) followed by Tukey’s tests was used for multiple groups’ comparison in GraphPad Prism (GraphPad Software, California, USA). s.e.m= standard error of the mean.

**Descriptive statistics:**

|  | **Sham** | s.e.m | **Injured** | s.e.m | **Injured+ sheath transplantation** | s.e.m |
| --- | --- | --- | --- | --- | --- | --- |
| Peak force (N) | 7.45 | 0.29 | 4.68 | 0.42 | 8.30 | 0.50 |
| Peak stress (N/mm^2^) | 3.88 | 0.21 | 0.98 | 0.07 | 1.81 | 0.10 |
| Stiffness (N/mm) | 8.57 | 0.53 | 6.26 | 0.52 | 8.43 | 0.56 |

**Tukey's multiple comparisons test (Adjusted P Value):**

|  | Peak force (N) | Peak stress (N/mm^2^) | Stiffness (N/mm) |
| --- | --- | --- | --- |
| Sham Vs. Injured | 0.0004 | <0.0001 | 0.0181 |
| Sham Vs. Injured+ sheath transplantation | 0.3464 | <0.0001 | 0.9806 |
| Injured Vs. Injured+ sheath transplantation | <0.0001 | 0.0010 | 0.0228 |
