# Supplementary material for: Osteocalcin expressing cells from tendon sheaths in mice contribute to tendon repair by activating Hedgehog signaling
Source: eLife. 2017 Dec 15;6:e30474. doi: 10.7554/eLife.30474 (PMC5731821; doi:10.7554/eLife.30474)
Supplement: Figure 3—source data 5. [file elife-30474-fig3-data5.docx]

| Gene | **Sheath control** | s.e.m | **GFP+ cells collected from injured tendon** | s.e.m | P-value | P-value summary |
| --- | --- | --- | --- | --- | --- | --- |
| *Mkx* | 1.17 | 0.29 | 2.61 | 0.18 | 0.0125 | * |
| *Scx* | 1.22 | 0.35 | 1.56 | 0.20 | 0.5100 | ns |
| *Col1a1* | 1.21 | 0.34 | 2.48 | 0.22 | 0.0383 | * |
| *Col1a2* | 1.11 | 0.22 | 1.53 | 0.49 | 0.3988 | ns |

**Figure 3 – source data 5.** Source data relating to Figure 3K. QRT-PCR analysis of tendon progenitor markers *Mkx*, *Scx*, main ECM components *Col1a1* and *Col1a2* using primary GFP^+^ cells FACS-sorted from the nude mice injured Achilles tendons with *BGLAP-Cre;Rosa26^mT/mG^* sheath transplantation at Day 14 with expression normalized to *β-tubulin* and the sheath control group. n=3 biological replicates per group. Statistical comparisons were performed using a two-tailed Student’s t-test in GraphPad Prism (GraphPad Software, California, USA). s.e.m= standard error of the mean.
