# Supplementary material for: Osteocalcin expressing cells from tendon sheaths in mice contribute to tendon repair by activating Hedgehog signaling
Source: eLife. 2017 Dec 15;6:e30474. doi: 10.7554/eLife.30474 (PMC5731821; doi:10.7554/eLife.30474)
Supplement: Figure 4—source data 1. [file elife-30474-fig4-data1.docx]

**Figure 4 – source data 1.** Source data relating to Figure 4A. QRT-PCR analysis of tendon progenitor markers *Mkx*, *Scx* and *Egr1* using sheath tissues of adult wild-type mice two weeks after injury with expression normalized to *β-tubulin* and the sham group. n=4 biological replicates per group. Statistical comparisons were performed using a two-tailed Student’s t-test in GraphPad Prism (GraphPad Software, California, USA). s.e.m= standard error of the mean.

| Gene | **Sham** | s.e.m | **Injured** | s.e.m | P-value | P-value summary |
| --- | --- | --- | --- | --- | --- | --- |
| *Mkx* | 1.06 | 0.21 | 19.11 | 1.55 | <0.0001 | *** |
| *Scx* | 1.03 | 0.13 | 8.00 | 1.62 | 0.0051 | ** |
| *Egr1* | 1.04 | 0.17 | 23.57 | 4.07 | 0.0015 | ** |
