# Supplementary material for: Osteocalcin expressing cells from tendon sheaths in mice contribute to tendon repair by activating Hedgehog signaling
Source: eLife. 2017 Dec 15;6:e30474. doi: 10.7554/eLife.30474 (PMC5731821; doi:10.7554/eLife.30474)
Supplement: Figure 4—source data 2. [file elife-30474-fig4-data2.docx]

**Figure 4 – source data 2.** Source data relating to Figure 4B. QRT-PCR analysis of tendon ECM components *Col1a1, Col1a2, Tnmd* and *Thbs4* using sheath tissues of adult wild-type mice two weeks after injury with expression normalized to *β-tubulin* and the sham group. n=4 biological replicates per group. Statistical comparisons were performed using a two-tailed Student’s t-test in GraphPad Prism (GraphPad Software, California, USA). s.e.m= standard error of the mean.

| Gene | **Sham** | s.e.m | **Injured** | s.e.m | P-value | P-value summary |
| --- | --- | --- | --- | --- | --- | --- |
| *Col1a1* | 1.03 | 0.14 | 22.52 | 5.08 | 0.0055 | ** |
| *Col1a2* | 1.06 | 0.21 | 26.30 | 5.49 | 0.0037 | ** |
| *Tnmd* | 1.01 | 0.09 | 28.80 | 4.29 | 0.0006 | *** |
| *Thbs4* | 1.04 | 0.17 | 17.22 | 2.32 | 0.0004 | *** |
