# Supplementary material for: Osteocalcin expressing cells from tendon sheaths in mice contribute to tendon repair by activating Hedgehog signaling
Source: eLife. 2017 Dec 15;6:e30474. doi: 10.7554/eLife.30474 (PMC5731821; doi:10.7554/eLife.30474)
Supplement: Figure 4—source data 3. [file elife-30474-fig4-data3.docx]

**Figure 4 – source data 3.** Source data relating to Figure 4C. QRT-PCR analysis of Hh signalling ligands *Dhh, Ihh and Shh* using sheath tissues of adult wild-type mice one week after injury with expression normalized to *Gapdh* and the sham group. n=6 biological replicates per group. Statistical comparisons were performed using a two-tailed Student’s t-test in GraphPad Prism (GraphPad Software, California, USA). s.e.m= standard error of the mean.

| Gene | **Sham** | s.e.m | **Injured** | s.e.m | P-value | P-value summary |
| --- | --- | --- | --- | --- | --- | --- |
| *Dhh* | 1.06 | 0.17 | 1.25 | 0.13 | 0.3694 | ns |
| *Ihh* | 1.03 | 0.12 | 1.08 | 0.05 | 0.7266 | ns |
| *Shh* | 1.02 | 0.09 | 4.97 | 0.57 | <0.0001 | *** |
