# Supplementary material for: Osteocalcin expressing cells from tendon sheaths in mice contribute to tendon repair by activating Hedgehog signaling
Source: eLife. 2017 Dec 15;6:e30474. doi: 10.7554/eLife.30474 (PMC5731821; doi:10.7554/eLife.30474)
Supplement: Figure 4—source data 4. [file elife-30474-fig4-data4.docx]

**Figure 4 – source data 4.** Source data relating to Figure 4F. QRT-PCR analysis of Hh signalling effector *Gli1*, sheath markers *Tppp3* and *Bglap* using sheath tissues of adult wild-type mice two weeks after injury with expression normalized to *β-tubulin* and the sham group. n=4 biological replicates per group. Statistical comparisons were performed using a two-tailed Student’s t-test in GraphPad Prism (GraphPad Software, California, USA). s.e.m= standard error of the mean.

| **Gene** | **Sham** | s.e.m | **Injured** | s.e.m | P-value | P-value summary |
| --- | --- | --- | --- | --- | --- | --- |
| *Gli1* | 1.07 | 0.23 | 5.21 | 0.66 | 0.001 | *** |
| *Tppp3* | 1.02 | 0.13 | 16.46 | 3.09 | 0.0025 | ** |
| *Bglap* | 1.04 | 0.15 | 16.18 | 1.39 | <0.0001 | *** |
