# Supplementary material for: Osteocalcin expressing cells from tendon sheaths in mice contribute to tendon repair by activating Hedgehog signaling
Source: eLife. 2017 Dec 15;6:e30474. doi: 10.7554/eLife.30474 (PMC5731821; doi:10.7554/eLife.30474)
Supplement: Figure 5—source data 1. [file elife-30474-fig5-data1.docx]

**Figure 5 – source data 1.** Source data relating to Figure 5B. Histologic analysis of the thickness of the Tibialis anterior tendon sheath of the *Ptch1^c/+^* and *Ptch1^c/c^;BGLAP-Cre* mice at 1, 2 and 4 month old. n=at least 10 biological replicates per group per time point. Statistical comparisons were performed using a two-tailed Student’s t-test in GraphPad Prism (GraphPad Software, California, USA). The experiments shown here are representative of 3 independent experiments. s.e.m= standard error of the mean.

**The thickness(μm) of sheath tissues:**

|  | ***Ptch1^c/+^*** | s.e.m | ***Ptch1^c/c^;BGLAP-Cre*** | s.e.m | P-value | P-value summary |
| --- | --- | --- | --- | --- | --- | --- |
| 1 month | 10.99 | 0.31 | 45.73 | 1.73 | <0.0001 | *** |
| 2 month | 18.04 | 1.40 | 90.00 | 1.12 | <0.0001 | *** |
| 4 month | 19.57 | 1.04 | 73.55 | 2.03 | <0.0001 | *** |
