# Supplementary material for: Osteocalcin expressing cells from tendon sheaths in mice contribute to tendon repair by activating Hedgehog signaling
Source: eLife. 2017 Dec 15;6:e30474. doi: 10.7554/eLife.30474 (PMC5731821; doi:10.7554/eLife.30474)
Supplement: Figure 5—source data 2. [file elife-30474-fig5-data2.docx]

**Figure 5 – source data 2.** Source data relating to Figure 5E. QRT-PCR analysis of Hh signalling effector *Gli1* using the Tibialis anterior tendon sheath tissues of the *Ptch1^c/+^* and *Ptch1^c/c^;BGLAP-Cre* mice at 2 month old with expression normalized to *Gapdh* and the *Ptch1^c/+^* group. n=3 biological replicates per group. Statistical comparisons were performed using a two-tailed Student’s t-test in GraphPad Prism (GraphPad Software, California, USA). s.e.m= standard error of the mean.

| Gene | ***Ptch1^c/+^*** | s.e.m | ***Ptch1^c/c^;BGLAP-Cre*** | s.e.m | P-value | P-value summary |
| --- | --- | --- | --- | --- | --- | --- |
| *Gli1* | 1.01 | 0.11 | 11.42 | 0.92 | 0.0004 | *** |
