# Supplementary material for: Osteocalcin expressing cells from tendon sheaths in mice contribute to tendon repair by activating Hedgehog signaling
Source: eLife. 2017 Dec 15;6:e30474. doi: 10.7554/eLife.30474 (PMC5731821; doi:10.7554/eLife.30474)
Supplement: Figure 5—figure supplement 1—source data 1. [file elife-30474-fig5-figsupp1-data1.docx]

**Figure 5 figure supplement 1– source data 1.** Source data relating to Figure 5 figure supplement 1D. Fluorescent immunocytochemistry analysis of pH3^+^ sheath cells isolated from the *Ptch1^c/+^* and *Ptch1^c/c^;BGLAP-Cre* mice at 2 month old. n=6 biological replicates per group. Statistical comparisons were performed using a two-tailed Student’s t-test in GraphPad Prism (GraphPad Software, California, USA). The experiments shown here are representative of 3 independent experiments. s.e.m= standard error of the mean.

|  | ***Ptch1^c/+^*** | s.e.m | ***Ptch1^c/c^;BGLAP-Cre*** | s.e.m | P-value | P-value summary |
| --- | --- | --- | --- | --- | --- | --- |
| Percentage of pH3^+^ cells/ Visual field (%) | 0.0651 | 0.0089 | 0.1031 | 0.0084 | 0.0111 | * |
