# Supplementary material for: Osteocalcin expressing cells from tendon sheaths in mice contribute to tendon repair by activating Hedgehog signaling
Source: eLife. 2017 Dec 15;6:e30474. doi: 10.7554/eLife.30474 (PMC5731821; doi:10.7554/eLife.30474)
Supplement: Figure 6—source data 1. [file elife-30474-fig6-data1.docx]

**Figure 6 – source data 1.** Source data relating to Figure 6B. Histologic analysis of the thickness of the Tibialis anterior tendon sheath of the *Smo^c/c^* and *Smo^c/c^;BGLAP-Cre* mice at 2, 4 and 8 month old. n=at least 9 biological replicates per group per time point. Statistical comparisons were performed using a two-tailed Student’s t-test in GraphPad Prism (GraphPad Software, California, USA). The experiments shown here are representative of 3 independent experiments. s.e.m= standard error of the mean.

**The thickness(μm) of sheath tissues:**

|  | ***Smo^c/c^*** | s.e.m | ***Smo^c/c^;BGLAP-Cre*** | s.e.m | P-value | P-value summary |
| --- | --- | --- | --- | --- | --- | --- |
| 2 month | 15.13 | 0.35 | 6.31 | 0.34 | <0.0001 | *** |
| 4 month | 15.33 | 0.69 | 6.27 | 0.72 | <0.0001 | *** |
| 8 month | 17.37 | 0.34 | 10.00 | 0.63 | <0.0001 | *** |
