# Supplementary material for: Osteocalcin expressing cells from tendon sheaths in mice contribute to tendon repair by activating Hedgehog signaling
Source: eLife. 2017 Dec 15;6:e30474. doi: 10.7554/eLife.30474 (PMC5731821; doi:10.7554/eLife.30474)
Supplement: Figure 7—source data 1. [file elife-30474-fig7-data1.docx]

**Figure 7 – source data 1.** Source data relating to Figure 7E. Hydroxyproline assay analysis using the Tibialis anterior tendon fibers of the *Smo^c/c^* and *Smo^c/c^;BGLAP-Cre* mice at 4 weeks after injury. n=4 biological replicates per group. One-way analysis of variance (ANOVA) followed by Tukey’s tests was used for multiple groups’ comparison in GraphPad Prism (GraphPad Software, California, USA). s.e.m= standard error of the mean.

| Quantity of collagen content | **Sham (ug/mg tendon)** | s.e.m | **Injured (ug/mg tendon)** | s.e.m |
| --- | --- | --- | --- | --- |
| ***Smo^c/c^*** | 8.84 | 0.40 | 7.19 | 0.61 |
| ***Smo^c/c^;BGLAP-Cre*** | 5.76 | 0.15 | 1.87 | 0.02 |

**Descriptive statistics:**

**Tukey's multiple comparisons test (Adjusted P Value):**

|  | Adjusted P Value | Adjusted P Value summary |
| --- | --- | --- |
| *Smo^c/c^* Sham Vs. *Smo^c/c^* Injured | 0.0381 | * |
| *Smo^c/c^;BGLAP-Cre* Sham Vs.  *Smo^c/c^;BGLAP-Cre* Injured | <0.0001 | *** |
| *Smo^c/c^* Injured Vs.  *Smo^c/c^;BGLAP-Cre* Injured | <0.0001 | *** |
