# Supplementary material for: Osteocalcin expressing cells from tendon sheaths in mice contribute to tendon repair by activating Hedgehog signaling
Source: eLife. 2017 Dec 15;6:e30474. doi: 10.7554/eLife.30474 (PMC5731821; doi:10.7554/eLife.30474)
Supplement: Figure 7—figure supplement 1—source data 1. [file elife-30474-fig7-figsupp1-data1.docx]

**Figure 7 figure supplement 1– source data 1.** Source data relating to Figure 7 figure supplement 1A. QRT-PCR analysis of tendon progenitor markers *Mkx* and *Scx* using the Tibialis anterior tendon fibers of the *Smo^c/c^* and *Smo^c/c^;BGLAP-Cre* mice at 4 weeks after injury with expression normalized to *Gapdh* and the *Smo^c/c^* sham group. n=4 biological replicates per group. One-way analysis of variance (ANOVA) followed by Tukey’s tests was used for multiple groups’ comparison in GraphPad Prism (GraphPad Software, California, USA). s.e.m= standard error of the mean. Adjusted P Value is the P value using Tukey's test compared with *Smo^c/c^* injured group.

| *Mkx* | **Sham** | s.e.m | Adjusted P Value | **Injured** | s.e.m | Adjusted P Value |
| --- | --- | --- | --- | --- | --- | --- |
| ***Smo^c/c^*** | 1.01 | 0.07 | 0.0011 | 1.67 | 0.14 | - |
| ***Smo^c/c^;BGLAP-Cre*** | 1.01 | 0.08 | - | 0.68 | 0.04 | <0.0001 |

| *Scx* | **Sham** | s.e.m | Adjusted P Value | **Injured** | s.e.m | Adjusted P Value |
| --- | --- | --- | --- | --- | --- | --- |
| ***Smo^c/c^*** | 1.01 | 0.09 | 0.0106 | 1.51 | 0.06 | - |
| ***Smo^c/c^;BGLAP-Cre*** | 1.02 | 0.11 | - | 1.19 | 0.10 | 0.1133 |
