# Supplementary material for: Osteocalcin expressing cells from tendon sheaths in mice contribute to tendon repair by activating Hedgehog signaling
Source: eLife. 2017 Dec 15;6:e30474. doi: 10.7554/eLife.30474 (PMC5731821; doi:10.7554/eLife.30474)
Supplement: Figure 7—figure supplement 1—source data 2. [file elife-30474-fig7-figsupp1-data2.docx]

**Figure 7 figure supplement 1– source data 2.** Source data relating to Figure 7 figure supplement 1B. QRT-PCR analysis of tendon ECM components *Col1a1, Col1a2, Tnmd* and *Thbs4* using the Tibialis anterior tendon fibers of the *Smo^c/c^* and *Smo^c/c^;BGLAP-Cre* mice at 4 weeks after injury with expression normalized to *Gapdh* and the *Smo^c/c^* sham group. n=4 biological replicates per group. One-way analysis of variance (ANOVA) followed by Tukey’s tests was used for multiple groups’ comparison in GraphPad Prism (GraphPad Software, California, USA). s.e.m= standard error of the mean. Adjusted P Value is the P value using Tukey's test compared with *Smo^c/c^* injured group.

| *Col1a1* | **Sham** | s.e.m | Adjusted P Value | **Injured** | s.e.m | Adjusted P Value |
| --- | --- | --- | --- | --- | --- | --- |
| ***Smo^c/c^*** | 1.01 | 0.08 | <0.0001 | 5.74 | 0.37 | - |
| ***Smo^c/c^;BGLAP-Cre*** | 1.02 | 0.12 | - | 0.06 | 0.00 | <0.0001 |

| *Col1a2* | **Sham** | s.e.m | Adjusted P Value | **Injured** | s.e.m | Adjusted P Value |
| --- | --- | --- | --- | --- | --- | --- |
| ***Smo^c/c^*** | 1.05 | 0.19 | <0.0001 | 4.44 | 0.18 | - |
| ***Smo^c/c^;BGLAP-Cre*** | 1.03 | 0.13 | - | 0.16 | 0.01 | <0.0001 |

| *Tnmd* | **Sham** | s.e.m | Adjusted P Value | **Injured** | s.e.m | Adjusted P Value |
| --- | --- | --- | --- | --- | --- | --- |
| ***Smo^c/c^*** | 1.09 | 0.25 | <0.0001 | 5.75 | 0.31 | - |
| ***Smo^c/c^;BGLAP-Cre*** | 1.03 | 0.13 | - | 0.16 | 0.01 | <0.0001 |

| *Thbs4* | **Sham** | s.e.m | Adjusted P Value | **Injured** | s.e.m | Adjusted P Value |
| --- | --- | --- | --- | --- | --- | --- |
| ***Smo^c/c^*** | 1.04 | 0.16 | 0.0001 | 2.17 | 0.12 | - |
| ***Smo^c/c^;BGLAP-Cre*** | 1.03 | 0.14 | - | 0.53 | 0.03 | <0.0001 |
