# Supplementary material for: Osteocalcin expressing cells from tendon sheaths in mice contribute to tendon repair by activating Hedgehog signaling
Source: eLife. 2017 Dec 15;6:e30474. doi: 10.7554/eLife.30474 (PMC5731821; doi:10.7554/eLife.30474)
Supplement: Figure 8—source data 1. [file elife-30474-fig8-data1.docx]

| Gene | **Ad-GFP** | s.e.m | **Ad-Cre** | s.e.m | P-value | P-value summary |
| --- | --- | --- | --- | --- | --- | --- |
| *Gli1* | 1.05 | 0.24 | 5.27 | 0.18 | 0.0001 | *** |
| *Tppp3* | 1.01 | 0.08 | 2.16 | 0.27 | 0.0155 | * |
| *Bglap* | 1.01 | 0.12 | 1.64 | 0.14 | 0.0247 | * |
| *Mkx* | 1.05 | 0.23 | 12.18 | 1.16 | 0.0007 | *** |
| *Scx* | 1.02 | 0.14 | 1.56 | 0.11 | 0.0394 | * |
| *Col1a1* | 1.01 | 0.09 | 1.89 | 0.18 | 0.0120 | * |
| *Col1a2* | 1.00 | 0.06 | 3.25 | 0.34 | 0.0028 | ** |

**Figure 8 – source data 1.** Source data relating to Figure 8A. QRT-PCR analysis of Hh signalling effector *Gli1*, sheath markers *Tppp3* and *Bglap*, tendon progenitor markers *Mkx* and *Scx* and main ECM components *Col1a1* and *Col1a2* using primary *Ptch1^c/c^* sheath cells infected with GFP- or Cre-adenovirus with expression normalized to *β-tubulin* and the Ad-GFP group. n=3 biological replicates per group. Statistical comparisons were performed using a two-tailed Student’s t-test in GraphPad Prism (GraphPad Software, California, USA). s.e.m= standard error of the mean.
