# Supplementary material for: Osteocalcin expressing cells from tendon sheaths in mice contribute to tendon repair by activating Hedgehog signaling
Source: eLife. 2017 Dec 15;6:e30474. doi: 10.7554/eLife.30474 (PMC5731821; doi:10.7554/eLife.30474)
Supplement: Figure 8—source data 2. [file elife-30474-fig8-data2.docx]

| Gene | **Control** | s.e.m | **PM** | s.e.m | P-value | P-value summary |
| --- | --- | --- | --- | --- | --- | --- |
| *Gli1* | 1.03 | 0.17 | 5.83 | 0.79 | 0.0041 | ** |
| *Tppp3* | 1.01 | 0.08 | 2.75 | 0.47 | 0.0213 | * |
| *Bglap* | 1.07 | 0.25 | 2.20 | 0.13 | 0.0152 | * |
| *Mkx* | 1.03 | 0.17 | 7.69 | 0.40 | 0.0001 | *** |
| *Scx* | 1.00 | 0.04 | 1.68 | 0.15 | 0.0131 | * |
| *Col1a1* | 1.00 | 0.07 | 1.76 | 0.13 | 0.0061 | ** |
| *Col1a2* | 1.00 | 0.05 | 2.08 | 0.26 | 0.0159 | * |

**Figure 8 – source data 2.** Source data relating to Figure 8B. QRT-PCR analysis of Hh signalling effector *Gli1*, sheath markers *Tppp3* and *Bglap*, tendon progenitor markers *Mkx* and *Scx* and main ECM components *Col1a1* and *Col1a2* using sorted GFP^+^ primary sheath cells from *BGLAP-Cre;Rosa26^mT/mG^* treated with 1000nM Hh agonist purmorphamine(PM) with expression normalized to *β-tubulin* and the control group. n=3 biological replicates per group. Statistical comparisons were performed using a two-tailed Student’s t-test in GraphPad Prism (GraphPad Software, California, USA). s.e.m= standard error of the mean.
