# Supplementary material for: Osteocalcin expressing cells from tendon sheaths in mice contribute to tendon repair by activating Hedgehog signaling
Source: eLife. 2017 Dec 15;6:e30474. doi: 10.7554/eLife.30474 (PMC5731821; doi:10.7554/eLife.30474)
Supplement: Figure 8—source data 3. [file elife-30474-fig8-data3.docx]

| Gene | **Ad-GFP** | s.e.m | **Ad-Cre** | s.e.m | P-value | P-value summary |
| --- | --- | --- | --- | --- | --- | --- |
| *Tgfb1* | 1.02 | 0.15 | 4.25 | 0.18 | 0.0002 | *** |
| *Tgfb2* | 1.01 | 0.10 | 2.71 | 0.48 | 0.0260 | * |
| *Tgfb3* | 1.02 | 0.13 | 1.47 | 0.05 | 0.0295 | * |
| *Smad7* | 1.02 | 0.13 | 6.74 | 0.64 | 0.0009 | *** |

**Figure 8 – source data 3.** Source data relating to Figure 8C. QRT-PCR analysis of TGFβ/smad3 signalling components *Tgfb1*, *Tgfb2*, *Tgfb3* and *Smad7* using primary *Ptch1^c/c^* sheath cells infected with GFP- or Cre-adenovirus with expression normalized to *β-tubulin* and the Ad-GFP group. n=3 biological replicates per group. Statistical comparisons were performed using a two-tailed Student’s t-test in GraphPad Prism (GraphPad Software, California, USA). s.e.m= standard error of the mean.
