# Supplementary material for: Osteocalcin expressing cells from tendon sheaths in mice contribute to tendon repair by activating Hedgehog signaling
Source: eLife. 2017 Dec 15;6:e30474. doi: 10.7554/eLife.30474 (PMC5731821; doi:10.7554/eLife.30474)
Supplement: Figure 8—source data 4. [file elife-30474-fig8-data4.docx]

| Gene | **Control** | s.e.m | **PM** | s.e.m | P-value | P-value summary |
| --- | --- | --- | --- | --- | --- | --- |
| *Tgfb1* | 1.03 | 0.16 | 2.99 | 0.13 | 0.0007 | *** |
| *Tgfb2* | 1.02 | 0.16 | 1.86 | 0.12 | 0.0137 | * |
| *Tgfb3* | 1.01 | 0.09 | 1.62 | 0.09 | 0.0085 | ** |
| *Smad7* | 1.00 | 0.06 | 2.23 | 0.08 | 0.0002 | *** |

**Figure 8 – source data 4.** Source data relating to Figure 8D. QRT-PCR analysis of TGFβ/smad3 signalling components *Tgfb1*, *Tgfb2*, *Tgfb3* and *Smad7* using sorted GFP^+^ primary sheath cells from *BGLAP-Cre;Rosa26^mT/mG^* treated with 1000nM Hh agonist purmorphamine (PM) with expression normalized to *β-tubulin* and the control group. n=3 biological replicates per group. Statistical comparisons were performed using a two-tailed Student’s t-test in GraphPad Prism (GraphPad Software, California, USA). s.e.m= standard error of the mean.
