# Supplementary material for: Osteocalcin expressing cells from tendon sheaths in mice contribute to tendon repair by activating Hedgehog signaling
Source: eLife. 2017 Dec 15;6:e30474. doi: 10.7554/eLife.30474 (PMC5731821; doi:10.7554/eLife.30474)
Supplement: Figure 9—source data 1. [file elife-30474-fig9-data1.docx]

**Figure 9 – source data 1.** Source data relating to Figure 9A. Dual luciferase assay of *Mkx* activities using reporter plasmids with 0.8kb (pGL3-Mkxp8K) or 4kb (pGL3-Mkx4K) of *Mkx* promoter regions with SMAD2 or SMAD3 overexpression respectively in HEK293T cells. n=3 biological replicates per group. Two-way analysis of variance (ANOVA) followed by Tukey’s tests was used for multiple groups’ comparison in GraphPad Prism (GraphPad Software, California, USA). s.e.m= standard error of the mean.

|  | **pcDNA3.1** | s.e.m | **SMAD2-HA** | s.e.m | **Flag-SMAD3** | s.e.m |
| --- | --- | --- | --- | --- | --- | --- |
| pGL3 Basic | 1.00 | 0.02 | 2.17 | 0.31 | 5.12 | 0.17 |
| pGL3-Mkxp8K | 92.89 | 1.61 | 119.46 | 6.50 | 1395.42 | 77.95 |
| pGL3-Mkx4K | 9.55 | 0.42 | 21.70 | 0.63 | 297.50 | 15.91 |

**Descriptive statistics of luciferase activity (fold):**

**Tukey's multiple comparisons test:**

|  | Adjusted P Value | Adjusted P Value summary |
| --- | --- | --- |
| pGL3 Basic:pcDNA3.1 vs. pGL3 Basic:SMAD2-HA | > 0.9999 | ns |
| pGL3 Basic:pcDNA3.1 vs. pGL3 Basic:Flag-SMAD3 | > 0.9999 | ns |
| pGL3 Basic:pcDNA3.1 vs. pGL3-Mkxp8K:pcDNA3.1 | 0.3204 | ns |
| pGL3 Basic:pcDNA3.1 vs. pGL3-Mkxp8K:SMAD2-HA | 0.0986 | ns |
| pGL3 Basic:pcDNA3.1 vs. pGL3-Mkxp8K:Flag-SMAD3 | < 0.0001 | *** |
| pGL3 Basic:pcDNA3.1 vs. pGL3-Mkx4K:pcDNA3.1 | > 0.9999 | ns |
| pGL3 Basic:pcDNA3.1 vs. pGL3-Mkx4K:SMAD2-HA | 0.9997 | ns |
| pGL3 Basic:pcDNA3.1 vs. pGL3-Mkx4K:Flag-SMAD3 | < 0.0001 | *** |
| pGL3-Mkxp8K:pcDNA3.1 vs. pGL3-Mkxp8K:SMAD2-HA | 0.9980 | ns |
| pGL3-Mkxp8K:pcDNA3.1 vs. pGL3-Mkxp8K:Flag-SMAD3 | < 0.0001 | *** |
| pGL3-Mkxp8K:pcDNA3.1 vs. pGL3-Mkx4K:pcDNA3.1 | 0.4369 | ns |
| pGL3-Mkxp8K:SMAD2-HA vs. pGL3-Mkxp8K:Flag-SMAD3 | < 0.0001 | *** |
| pGL3-Mkxp8K:SMAD2-HA vs. pGL3-Mkx4K:SMAD2-HA | 0.2532 | ns |
| pGL3-Mkxp8K:Flag-SMAD3 vs. pGL3-Mkx4K:Flag-SMAD3 | < 0.0001 | *** |
| pGL3-Mkx4K:pcDNA3.1 vs. pGL3-Mkx4K:SMAD2-HA | > 0.9999 | ns |
| pGL3-Mkx4K:pcDNA3.1 vs. pGL3-Mkx4K:Flag-SMAD3 | < 0.0001 | *** |
| pGL3-Mkx4K:SMAD2-HA vs. pGL3-Mkx4K:Flag-SMAD3 | < 0.0001 | *** |
