# Supplementary material for: Osteocalcin expressing cells from tendon sheaths in mice contribute to tendon repair by activating Hedgehog signaling
Source: eLife. 2017 Dec 15;6:e30474. doi: 10.7554/eLife.30474 (PMC5731821; doi:10.7554/eLife.30474)
Supplement: Figure 9—source data 2. [file elife-30474-fig9-data2.docx]

**Figure 9 – source data 2.** Source data relating to Figure 9B. QRT-PCR analysis of TGFβ/smad3 signalling target gene *Smad7,* tendon progenitor marker *Mkx* and main ECM components *Col1a1* and *Col1a2* using sorted GFP^+^ primary sheath cells from *BGLAP-Cre;Rosa26^mT/mG^* treated with 1000nM Hh agonist purmorphamine(PM) with or without specific type I TGFβ receptor inhibitor SB431542 (10μM, 1 h) pre-treatment. The gene expression was normalized to β-tubulin and the control group. n=4 biological replicates per group. One-way analysis of variance (ANOVA) followed by Tukey’s tests was used for multiple groups’ comparison in GraphPad Prism (GraphPad Software, California, USA). s.e.m= standard error of the mean.

**(1) *Smad7***

|  | **Control** | s.e.m | **PM** | s.e.m |
| --- | --- | --- | --- | --- |
| Control | 1.03 | 0.16 | 3.31 | 0.34 |
| SB431542 | 1.09 | 0.16 | 1.87 | 0.24 |

Descriptive statistics:

Tukey's multiple comparisons test:

|  | Adjusted P Value | Adjusted P Value summary |
| --- | --- | --- |
| Control Vs. PM | <0.0001 | *** |
| Control Vs. SB431542 | 0.9978 | ns |
| Control Vs. PM+ SB431542 | 0.1065 | ns |
| PM Vs. SB431542 | 0.0001 | *** |
| PM Vs. PM+ SB431542 | 0.0045 | ** |
| SB431542 Vs. PM+ SB431542 | 0.1429 | ns |

**(2) *Mkx***

Descriptive statistics:

|  | **Control** | s.e.m | **PM** | s.e.m |
| --- | --- | --- | --- | --- |
| Control | 1.01 | 0.09 | 8.16 | 0.64 |
| SB431542 | 1.14 | 0.10 | 1.82 | 0.17 |

Tukey's multiple comparisons test:

|  | Adjusted P Value | Adjusted P Value summary |
| --- | --- | --- |
| Control Vs. PM | <0.0001 | *** |
| Control Vs. SB431542 | 0.9921 | ns |
| Control Vs. PM+ SB431542 | 0.3631 | ns |
| PM Vs. SB431542 | <0.0001 | *** |
| PM Vs. PM+ SB431542 | <0.0001 | *** |
| SB431542 Vs. PM+ SB431542 | 0.5078 | ns |

**(3) *Col1a1***

|  | **Control** | s.e.m | **PM** | s.e.m |
| --- | --- | --- | --- | --- |
| Control | 1.02 | 0.12 | 1.70 | 0.09 |
| SB431542 | 0.59 | 0.09 | 0.56 | 0.07 |

Descriptive statistics:

Tukey's multiple comparisons test:

|  | Adjusted P Value | Adjusted P Value summary |
| --- | --- | --- |
| Control Vs. PM | 0.0012 | ** |
| Control Vs. SB431542 | 0.0293 | * |
| Control Vs. PM+ SB431542 | 0.0188 | * |
| PM Vs. SB431542 | <0.0001 | *** |
| PM Vs. PM+ SB431542 | <0.0001 | *** |
| SB431542 Vs. PM+ SB431542 | 0.9941 | ns |

**(4) *Col1a2***

|  | **Control** | s.e.m | **PM** | s.e.m |
| --- | --- | --- | --- | --- |
| Control | 1.02 | 0.11 | 2.06 | 0.19 |
| SB431542 | 0.67 | 0.06 | 0.85 | 0.09 |

Descriptive statistics:

Tukey's multiple comparisons test:

|  | Adjusted P Value | Adjusted P Value summary |
| --- | --- | --- |
| Control Vs. PM | 0.0003 | *** |
| Control Vs. SB431542 | 0.2408 | ns |
| Control Vs. PM+ SB431542 | 0.7817 | ns |
| PM Vs. SB431542 | <0.0001 | *** |
| PM Vs. PM+ SB431542 | <0.0001 | *** |
| SB431542 Vs. PM+ SB431542 | 0.7214 | ns |
