# Supplementary material for: Osteocalcin expressing cells from tendon sheaths in mice contribute to tendon repair by activating Hedgehog signaling
Source: eLife. 2017 Dec 15;6:e30474. doi: 10.7554/eLife.30474 (PMC5731821; doi:10.7554/eLife.30474)
Supplement: Supplementary file 1. [file elife-30474-supp1.docx]

Supplementary File 1 Primer sequences for QRT-PCR

| Gene name | Sequences |
| --- | --- |
| *β-tubulin* | forward: 5′- GTGCACATTCAGGCGGGCCA -3′  reverse: 5′- GCCTGGCTCCAGGTCCACCA -3′ |
| *Tppp3* | forward: 5′- GCTGGCAACTAAGCGGTTCAAG -3′  reverse: 5′- ACTTACTGGTGTCCGTCAGCCG -3′ |
| *Bglap* | forward: 5′- GCAATAAGGTAGTGAACAGACTCC -3′  reverse: 5′- CCATAGATGCGTTTGTAGGCGG -3′ |
| *Dmp1* | forward: 5′- AGAGGAATCGCATCCCAAT -3′  reverse: 5′- ACCCAGCCAAATCACCCG -3′ |
| *Ibsp1* | forward: 5′- AAAATGGAGACGGCGATAG -3′  reverse: 5′- TTCTTGGGCAGTTGGAGTG -3′ |
| *Mepe* | forward: 5′- ACTAAGCCCGAAGAAGCC -3′  reverse: 5′- CACAGATGCTGCCAAGTC -3′ |
| *Pparγ* | forward: 5′- AGCTGACCCAATGGTTGCTG -3′  reverse: 5′- CCTTGCATCCTTCACAAGCATG -3′ |
| *Cebpa* | forward: 5′- CTTACAACAGGCCAGGTTTCC -3′  reverse: 5′- CCTCTGGGATGGATCGATTGT -3′ |
| *Fabp4* | forward: 5′- TGGAAGCTTGTCTCCAGTGAAAA -3′  reverse: 5′- GAAGTCACGCCTTTCATAACACAT -3′ |
| *Lpl* | forward: 5′- AGTCTGGCTGACACTGGACAA -3′  reverse: 5′- GCTATGGGCCATTAGATTCCTCAC -3′ |
| *Sox9* | forward: 5′- CAGGTGCTGAAGGGCTAC -3′  reverse: 5′- ​GGAGGAATGTGGGGAGTC -3′ |
| *Aggrecan* | forward: 5′- TCCCCAAATCCCTCATAC -3′  reverse: 5′- TAGTCCACCCCTCCTCAC -3′ |
| *Col2a1* | forward: 5′- GACTGAAGGGACACCGAG -3′  reverse: 5′- CCAGGGATTCCATTAGAG -3′ |
| *Mkx* | forward: 5′- TGGTTTCCTGGACAATCCACA -3′  reverse: 5′-CGCTTATGCCTTACCTTCCCTC -3′ |
| *Scx* | forward: 5′- ACCGCACCAACAGCGTGAACAC -3′  reverse: 5′- CAGCACATTGCCCAGGTGAGAA -3′ |
| *Egr1* | forward: 5′- CAGCGCCTTCAATCCTCAAG -3′  reverse: 5′- GCGATGTCAGAAAAGGACTCTGT -3′ |
| *Tnmd* | forward: 5′- ACACTTCTGGCCCGAGGTAT -3′  reverse: 5′- GACTTCCAATGTTTCATCAGTGC -3′ |
| *Thbs4* | forward: 5′- TCCACGTAAACACCCAGACA -3′  reverse: 5′- TTCTGCTACTGCACGGAATG -3′ |
| *Col1a1* | forward: 5′- CCCAATGGTGAGACGTGGAA -3′  reverse: 5′- TTGGGTCCCTCGACTCCTAC -3′ |
| *Col1a2* | forward: 5′- CAGGTGCCAGAGGACTTGTT -3′  reverse: 5′- GCCAGGAGGACCCATTACAC -3′ |
| *Runx2* | forward: 5′- AACTTCCTGTGCTCCGTG -3′  reverse: 5′- CGTTGAACCTGGCTACTT -3′ |
| *Sp7* | forward: 5′- GGCTTTTCTGCGGCAAGAGGTT -3′  reverse: 5′- CGCTGATGTTTGCTCAAGTGGTC -3′ |
| *Gli1* | forward: 5′- GCTGGTGGTGCACATGCGCAG -3′  reverse: 5′- GTGTAGCGCTTGGTGCAGCC -3′ |
| *Tgfb1* | forward: 5′- TGATACGCCTGAGTGGCTGTCT -3′  reverse: 5′- CACAAGAGCAGTGAGCGCTGAA -3′ |
| *Tgfb2* | forward: 5′- TTGTTGCCCTCCTACAGACTGG -3′  reverse: 5′- GTAAAGAGGGCGAAGGCAGCAA -3′ |
| *Tgfb3* | forward: 5′- AAGCAGCGCTACATAGGTGGCA -3′  reverse: 5′- GGCTGAAAGGTGTGACATGGAC -3′ |
| *Tgfbr1* | forward: 5′- TGCTCCAAACCACAGAGTAGGC -3′  reverse: 5′- CCCAGAACACTAAGCCCATTGC -3′ |
| *Tgfbr2* | forward: 5′- CCTACTCTGTCTGTGGATGACC -3′  reverse: 5′- GACATCCGTCTGCTTGAACGAC -3′ |
| *Smad7* | forward: 5′- GTCCAGATGCTGTACCTTCCTC -3′  reverse: 5′- GCGAGTCTTCTCCTCCCAGTAT -3′ |
